# Overcoming Confounding to Characterize the Effects of Calcium Channel Blockers

**DOI:** 10.1093/function/zqad054

**Published:** 2023-10-12

**Authors:** Sudarshan Rajagopal, Paul B Rosenberg

**Affiliations:** Division of Cardiology, Department of Medicine, Duke University Medical Center, Durham, NC, 27710, USA; Division of Cardiology, Department of Medicine, Duke University Medical Center, Durham, NC, 27710, USA

## A Perspective on “A Reappraisal of the Effects of L-type Ca^2+^ Channel Blockers on Store-operated Ca^2+^ Entry and Heart Failure”

Hypertension is the leading cause of death globally, accounting for 10.4 million deaths annually, with long-term effects that include an increased risk of myocardial infarction, heart failure, stroke, retinopathy, and kidney disease.^1^ Current therapies for hypertension include calcium channel blockers (CCBs), angiotensin-converting enzyme inhibitors (ACEIs), angiotensin receptor blockers (ARBs), thiazide diuretics, and beta blockers.^1^ Of these, CCBs are now one of the most popular and are currently first-line agents, with numerically greater reductions in blood pressure in certain patient groups.^2^ One of the most popular is the dihydropyridine CCB amlodipine, which can be taken daily, is effective, and has relatively few side effects. Recently, Johnson et al.^3^ have raised questions regarding the safety of amlodipine and CCBs in general, as they reported that all CCBs activated store-operated Ca^2+^ entry (SOCE) in addition to their well-established role in the inhibition of voltage-operated Ca^2+^ channels. Moreover, they observed that CCBs stimulated SOCE in rat aortic smooth muscle cells and promoted greater proliferation, which would be expected to have negative impacts on vascular function. These findings are consistent with previous studies where blockade or genetic deletion of SOCE was shown to impair vascular smooth muscle cell proliferation, potentially impacting several vascular diseases.^4^ In fact, genetic variation in the STIM1 gene was identified as a modifier gene for the spontaneously hypertensive rat.^5^ Understanding the actions of CCB on SOCE therefore has implications on many vascular diseases. Consistent with this idea, Johnson et al. also performed an epidemiological analysis of ∼20 000 patient records that suggested that CCBs increased the risk of heart failure. This finding was surprising as it was counter to the many clinical trials that underpin current clinical guidelines and runs counter to the clinical experience of many hypertension and heart failure specialists. However, if these findings were true, it would suggest that CCBs should not be first-line agents for hypertension and that their use in general should be revisited.

This puts into the context of Bird et al.,^6^ who sought to rigorously test the effects of amlodipine and CCBs on SOCE as well as testing their clinical effects. One of the major differences from the present study and Johnson et al.^3^ was the choice of Ca^2+^-sensitive fluorescent dyes that were used to measure cytosolic calcium. In the study by Johnson et al., fura-2 was used to measure Ca^2+^ signals. However, Bird et al. found that amlodipine besylate interfered with the interpretation of signal from fura-based dyes, as amlodipine and fura-2 have overlapping excitation spectra. Moreover, they found that amlodipine accumulated within the cytoplasm over several minutes of exposure, dominating the fluorescence signal in fura-2–loaded cells, and thereby mimicking a Ca^2+^ transient. This is consistent with previous studies that amlodipine can cross the plasma membrane and accumulate in intracellular vesicles.^7^ For this reason, Bird et al. used the longer wavelength dye Cal-520 to monitor cytosolic Ca^2+^ in their studies with dihydropyridine CCBs. With this reporter, they found no evidence for direct activation of Ca^2+^ release-activated Ca^2+^ (CRAC) channels by these drugs. This study highlights the importance of rigorously testing any hypothesis with different techniques or approaches.

However, there were more to the findings than simply demonstrating amlodipine artifactually effected Ca^2+^ transients that likely confounded the interpretation of the earlier study. The authors report complex actions of amlodipine on Ca^2+^ release and SOCE. Amlodipine can release thapsigargin-sensitive stores, limit SOCE, but has no effect of nuclear factor of activated T cells (NFAT) nuclear translocation. These findings are unexpected as agents that promote the release of internal stores would be expected to activate the Ca^2+^ sensor stromal interaction molecule 1 (STIM1) and trigger SOCE. Amlodipine also inhibited CRAC channels, thus preventing SOCE despite store depletion. The authors also found that CCBs have complex and multimodal actions on SOCE, with different CCBs having very different effects: Nifedipine had no effect on SOCE, while diltiazem activated the channels and amlodipine inhibited them. However, at therapeutic concentrations, none of the CCBs activated SOCE. In many of the studies performed here^6^ and in Johnson et al.,^3^ the concentrations ranged from 1 to 10 μm, much higher than the typical therapeutic levels of amlodipine in the range of 0.73-36 nm.^8^Thus, it is likely that the only effects for these drugs at therapeutic levels would be the inhibition of Cav1.2 channels, with no effect on CRAC channels.

The effects of amlodipine on vascular smooth muscle cells were not tested here, as Johnson et al.^3^ had shown that amlodipine promoted their proliferation, although earlier studies have suggested that dihydropyridines inhibit vascular myocyte proliferation.^9^ Notably, Bird et al. did not detect any activation of NFAT-dependent gene expression, which is well established as a downstream target of SOCE, and would suggest that vascular myocyte proliferation in response to amlodipine is unlikely. Together, these findings suggest that amlodipine is unlikely to promote vascular smooth muscle cell proliferation.

Lastly, the authors complemented their rigorous wet lab studies with an updated meta-analysis and network meta-analysis that confirmed that dihydropyridine CCBs are protective against incident heart failure. This was complemented by a real-world analysis of over 63 000 patients without prior cardiovascular disease who were started on a single agent of 1 of 5 antihypertensive classes: dihydropyridine CCBs, nondihydropyridine CCBs, ACEI/ARB, thiazides, and beta blockers. While such real-world data can be confounded, a reduced risk of incident heart failure compared to ACEI/ARB and beta blocker was observed. These findings are all consistent with current guidelines that recommend amlodipine as a first-line agent for hypertension.^1^

Thanks to the rigorous studies in this work, we now have a better understanding of the true effects of amlodipine and CCBs on SOCE, and the clinical utility and safety of amlodipine is no longer in question.
